# Viable *Leishmania* parasites in the absence of an *in vitro* IFN-γ response in asymptomatic carriers

**DOI:** 10.1590/S1678-9946202466013

**Published:** 2024-02-19

**Authors:** Elsy Nalleli Loría-Cervera, Erika Ivett Sosa-Bibiano, Karina Beatriz López-Ávila, Ana Celia Montes de Oca-Aguilar, Marisol Sarahí Moreno-Nava, Jimmy Raymundo Torres-Castro

**Affiliations:** 1 Universidad Autónoma de Yucatán, Centro de Investigaciones Regionales “Dr. Hideyo Noguchi”, Laboratorio de Inmunología, Mérida, Yucatán, Mexico; 2 Servicios de Salud del Estado de Yucatán, Dirección de Prevención y Protección de la Salud, Mérida, Yucatán, Mexico

**Keywords:** Leishmaniasis, Cutaneous leishmaniasis, Asymptomatic infection, Immune response, IFN-γ expression

## Abstract

Asymptomatic infection (the absence or inapparent signs and symptoms) has been observed in many endemic areas of leishmaniasis, however, little is known about the parasitological and immunological factors associated with this type of infection. This study aimed to identify the *in vitro* expression of IFN-γ in asymptomatic carriers of viable *Leishmania* parasites. Asymptomatic infection was identified using the Montenegro skin test in an at-risk population from Yucatan, Mexico. Parasite viability was evinced in the blood by 7SL RNA transcripts amplification. The expression of mRNA IFN-γ was analyzed in peripheral blood mononuclear cells stimulated with soluble *Leishmania* antigen, using RT-qPCR. Parasite viability was observed in 33.3 % (5/15) of asymptomatic subjects. No differences were found in the expression of IFN-γ between asymptomatic and healthy subjects, and no correlation was found between the presence of viable parasites and the expression of IFN-γ. This study demonstrates the persistence of *Leishmania* parasites in the absence of an *in vitro* IFN-γ response in asymptomatic carriers from Mexico.

## INTRODUCTION

Leishmaniasis is a parasitic disease that causes cutaneous or visceral manifestations that can be disabling and lead to severe social stigma, especially in neglected populations^1^. Asymptomatic *Leishmania* infection is by far the most common outcome in endemic areas^2^. Asymptomatic individuals are those who live in *Leishmania*-endemic areas and do not show signs/symptoms of the disease but are positive to serological, molecular, cellular, or parasitological tests^3^. Studying the asymptomatic population is vital, as it can serve as a parasite reservoir and represent an epidemiological risk. In addition, it is considered a resistant population capable of controlling *Leishmania* infection^3,4^.

The Montenegro skin test (MST) is one of the most useful markers for asymptomatic infection and evokes a classic T-cell mediated inflammatory reaction known as the delayed-type hypersensitivity (DTH) response^3^. The absence of signs and symptoms in asymptomatic subjects has been attributed to the high production of IFN-γ, a Th1 cytokine associated with the resolution of *Leishmania* infection and frequently used as a marker of asymptomatic infection^4,5^. However, contrasting results have been obtained about its value in identifying asymptomatic populations, since in humans, the production of IFN-γ seems to be associated with the infecting *Leishmania* species and exposure time, among other factors^6,7^. On the other hand, persistent *Leishmania* infection in asymptomatic subjects has recently been demonstrated by the amplification of the parasite’s 7SL RNA gene transcripts, which provides evidence of their potential contribution to the endemicity and transmission of the disease^8^. However, to our knowledge, there are no studies linking the production of IFN-γ to the presence of live parasites in asymptomatic carriers.

This study aims to evaluate the IFN-γ expression in peripheral blood mononuclear cells (PBMC) stimulated with soluble *Leishmania* antigen in asymptomatic subjects with evidence of viable parasites in their blood. This is a preliminary exploration to explain the parasitological and immunological features that regulate asymptomatic infections caused by *Leishmania mexicana*.

## MATERIALS AND METHODS

### Study design, study population, and ethics statement

From November 2019 to February 2020, a cross-sectional study design was employed to identify asymptomatic *Leishmania* infection in a convenience sample according to known risk factors for cutaneous leishmaniosis (CL) in the Yucatan Peninsula^9^. This study included individuals of both sexes, aged 15 to 90 years, who had carried out activities in the tropical forest of the Tinum municipality, Yucatan and could therefore have been exposed to the bite of Phlebotomine sand flies^9^. The MST was applied after an interview to collect demographic data. During the interview, skin surfaces were examined to ensure the absence of typical scars or active lesions caused by *Leishmania* ^9^. Derived from this active epidemiological survey for the detection of asymptomatic infection, the MST was applied to 74 subjects who met the aforementioned criteria, in an emerging focus of CL in the Yucatan State.

The leishmanin used for the MST was prepared with 10^6^ promastigotes of an authochtonous strain of *L. mexicana* (MHET/MX/97/Hd18) in 0.05% phenol saline; 0.04 mL of the solution was injected intradermally into the left forearm. For this purpose, promastigotes were grown in Tc medium for seven days at 23 °C. Stationary phase promastigotes were washed trice in RPMI-1640 (RPMI medium, Gibco) before being counted and adjusted to the concentrations needed for the preparation of leishmanin. The skin reaction was assessed 48 h later using the ballpoint pen method^9^. The test was considered positive if the mean of the two diameters was 5 mm or more. An erythematous response alone was recorded as negative.

This study was approved by the Research Ethics Committee of the Autonomous University of Yucatan under ID CEI-22-2018. Written informed consent was obtained from each participant aged over 18 years after explaining the purpose of the study. Written informed consent was obtained from the guardians of participants aged 15 to 17 years, as well as written assent from these participants.

### Detection of Leishmania RNA in the blood

*Leishmania* RNA was detected in the blood of the 15 asymptomatic individuals with a positive MST who agreed to take part in the second phase of the study. Total RNA was extracted from 100 μl of whole blood using TRIzol reagent (SIGMA), following the manufacturer’s instructions. RNA integrity was monitored by electrophoresis in 2% agarose gel containing ethidium bromide (0.5 µg/mL). Total RNA (200 ng) was reverse transcribed into cDNA using the Improm-II reverse transcription system kit (Promega), following the manufacturer’s instructions. As a quality control for RNA integrity, the 18S gene was amplified using consensus primers and previously reported conditions^10^. Real-time PCR was performed to amplify a 173 bp fragment of the 7SL RNA gene from the *Leishmania* genus using the commercial kit iTaq Universal SYBR Green Supermix (Biorad) and 1 μM of the following primers: TRY7SL.For1.M13 (5-GTA AAA CGA CGG CCA GTG CTC TGT AAC CTT CGG GGG CT-3) and TRY7SL.Rev1.M13 (5-CAG GAA ACA GCT ATG ACG GCT GCT CCG TYN CCG GCC TGA CCC-3)^11^. This target is an integral component of the signal recognition particle that mediates protein translocation across the endoplasmic reticulum. Both its abundance in the cytoplasm and its short half-life support the feasibility of sensitive detection of live *Leishmania* parasites in the samples^11^. PCR amplification was performed in a StepOne thermocycler (Applied Biosystems) with an initial denaturation step at 95 °C for 2 min, followed by 40 cycles of 95 °C for 15 s and 67 °C for 1 min. In the final cycle, a melting curve was obtained at 95 °C for 15 s, 67 °C for 1 min, and 95 °C for 15 s. The cDNA from uninfected human blood and the cDNA obtained from *L. mexicana* parasites in culture were included as negative and positive controls, respectively.

### Sequencing, alignment, similarity, and phylogenetic analysis

The 7SL RNA amplicons were obtained by conventional PCR using the conditions described above, except for the fact that the Taq ReadyMix^TM^ (SIGMA P4600) was used. The fragments were directly sequenced using the ABI PRISM System (Applied Biosystems) of Big Dye fluorescent terminators and the ABI 310 capillary electrophoresis instrument. Sequencing and PCR amplification procedures were performed using the same primer sets. The sequences were edited using Sequencher v. 4.1.4 (Gene Codes Corp., Ann Arbor, Michigan, USA) and subsequently aligned using Mega v.11.0.11 (Molecular Evolutionary Genetics Analysis). A manual alignment was used following the similarity criterion proposed by Simmons^12^. The sequences were compared using BLAST (Basic Local Alignment Search Tool). The expected value, percentage of identity, query cover, and total score were considered for the analysis.

Phylogenetic analyses were conducted using a consensus sequence. GenBank sequences of 12 species of *Leishmania* parasite were used for the analysis. A *Trypanosoma brucei* sequence was used as an outgroup. The best nucleotide substitution model was estimated and selected in jModelTest v.2.1.6 using the Akaike information criterion (AICc). Phylogenetic estimation was conducted using the Maximum likelihood (ML) approach. A machine learning (ML) analysis was run in RAxML v.8 using the CIPRES Science Gateway. Node support for the ML tree was estimated with 1,000 bootstrap replicates, and nodes were considered highly supported when bootstrap values were > 70%. Additionally, a dendrogram was created in Mega v.11.0.11 using the neighbor-joining method, and node support was also estimated with 2,500 bootstrap replicates^13^.

### Isolation of peripheral blood mononuclear cells and expression analysis of IFN-γ

For the expression analysis of IFN-γ, samples from 11 asymptomatic individuals, which passed the integrity quality control with the constitutive 18S gene, were included. To compare the *in vitro* expression levels of this cytokine, nine healthy non-endemic controls with negative MST results—which, given these results, had never been exposed to the *Leishmania* parasite—were included.

PBMC were collected by centrifugation on a Ficoll-Paque PREMIUM gradient (GE Healthcare Bio-Sciences Corp). After washing three times with PBS, the PBMC were resuspended in RPMI-1640 medium (GIBCO) supplemented with 10% fetal bovine serum (FBS), 0.1% β-mercaptoethanol, 10 mM L-glutamine, 20 mM sodium pyruvate, and 100 U/ml penicillin-streptomycin. Live cells were adjusted to 1x10^6^ cells/mL, placed in 24-well plates, and stimulated with soluble *Leishmania* antigen (SLA) at 10 µg/mL for 72 h at 37 °C and 5% CO_2._

For the preparation of the SLA, the *L. (L.) mexicana* MHET/MX/97/Hd18 strain was selected and its infectivity was restored by passage in Syrian golden hamsters. Promastigotes were grown in Tc medium for seven days at 23 °C. SLA was obtained from stationary promastigotes, which were washed twice in phosphate buffered saline (PBS), resuspended in PBS and phenylmethanesulfonyl fluoride, and subjected to five freeze-thaw cycles at −70 °C and 37 °C. The protein content was determined using the Bradford method.

The stimulated cells were separated by pipetting and recovered by centrifugation. RNA was extracted using TRIzol Reagent (SIGMA). RNA samples (100 ng) were treated with DNase (1 U/μL) and reverse transcribed using the Improm-II^TM^ Reverse Transcription system kit (Promega). The expression of IFN-γ was determined using the iTaq Universal SYBR Green Supermix (Biorad) and a final concentration of 50 nM of the primers FW (5-TCGGTAACTGACTTGAATGTCCA-3) and RV (5-TCGCTTCCCTGTTTTAGCTGC-3)^14^ in an StepOne equipment (Applied Biosystems). The PCR conditions were 95 °C for 30 s, followed by 40 cycles at 94 °C for 10 s, 60 °C for 18 s, and 72 °C for 20 s. At the end of the reaction, a melting curve was obtained at 95 °C for 10 s, 60 °C for 18 s, and 95 °C for 10 s. The cDNA of cells incubated with Concanavalin A (5 µg/mL) or with the medium alone were included as positive and negative controls, respectively. The relative quantification of IFN-γ in the samples and controls was estimated with the 2-ΔΔCT method, using 18S rRNA as an endogenous control. The data were presented as the fold change in gene expression relative to the unstimulated cells.

### Statistical analysis

Descriptive statistics were used to present the demographic data. For the analysis of mRNA expression, the Kolmogorov-Smirnov test was used to examine data normality. None of the groups passed the normality test, so IFN-γ expression between the asymptomatic groups and non-endemic controls was compared using the Kruskal-Wallis test. The data were analyzed using GraphPad Prism 5.0 (GraphPad Software, Inc., San Diego, CA, USA).

A linear regression was performed to assess the relationship between IFN-γ expression and the presence of viable *Leishmania* parasites. The model was run with the lm4 package, and the explained variance was expressed by R^2.^ The fitted models were checked by plotting the standardized residuals against fitted values using the DHARMA package. The models were built in R v. 4. 2. 1.

## RESULTS

The MST was applied to 74 subjects with risk factors for *Leishmania* infection. The test was positive for 20 of them (27 %), and induration sizes ranged from 5 to 17.5 mm (x^–^ = 10.2 mm). Most asymptomatic subjects were male (80%), with a mean age of 59.7 years (range 15–87 years). Regarding activities that put them at risk, 45 % of them were farmers, 15 % were wood collectors, and 40% conducted both activities. The time they spent in the dry forest surrounding their locations—that is, their exposure time—ranged from 1 to 12 h.

**Figure 1:**
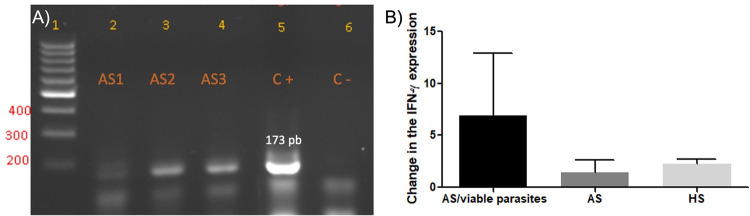
A) Representative agarose gel electrophoresis of the PCR products of *Leishmania* 7SL RNA gene from the blood of asymptomatic subjects. Row 1: molecular marker (100 pb). Rows 2-4: samples from asymptomatic subjects (AS). Row 5: positive cDNA control obtained from parasites in culture. Row 6: negative control (reaction mix without cDNA); B) Expression analysis of IFN-γ from PBMCs stimulated with ASL in asymptomatic and control groups. Data are shown as mean and standard error.

Parasite viability was evidenced by the detection of 7SL RNA transcripts in 33.3 % (5/15) of the asymptomatic subjects (Figure 1A). Most asymptomatic subjects positive for the presence of viable *Leishmania* parasites in the blood were male (80%, 4/5). One of the four women tested was positive for the 7SL RNA transcript (25%). The mean age of the subjects positive for viable parasites in the blood was 67.8 (range 48–78 years) and their induration sizes ranged from 7 to 14 mm (x^–^ = 11.9, SD= 2.97, mode = 14).

No significant differences (p = 0.3748) in IFN-γ expression were found among asymptomatic subjects with viable parasites, asymptomatic subjects without evidence of parasites in their blood and non-endemic controls (Figure 1B). The fold change in IFN-γ expression in subjects with evidence of *Leishmania* RNA in their blood was variable and ranged from 0.07 to 30.71 (x^–^ = 6.91). The mean fold change was 369 ±657 (ED) in the cells incubated with ConA (positive control). Non-significant associations were found between the presence of viable *Leishmania* parasites in the blood and the expression of IFN-γ *in vitro* (Table 1).

**Table 1:** Results of the linear regression analysis in which the individual is considered a random variable, the changes in IFN-γ expression (C_IFN) are considered the dependent variable, and live parasites in the blood (LPB) are considered an independent variable.

Model	Component	Estimate	Std. error	t value	Pr(>|t|)	Adjusted R^2^
Null model	Intercept	10.8	0.9744	11.08	2.58E-08	–
Model 1	Intercept	10.2	1.204	8.468	1.19E-06	−0.05
	ChangesExpIFN~LPB	1.8	2.086	0.863	0.404	

**Figure 2:**
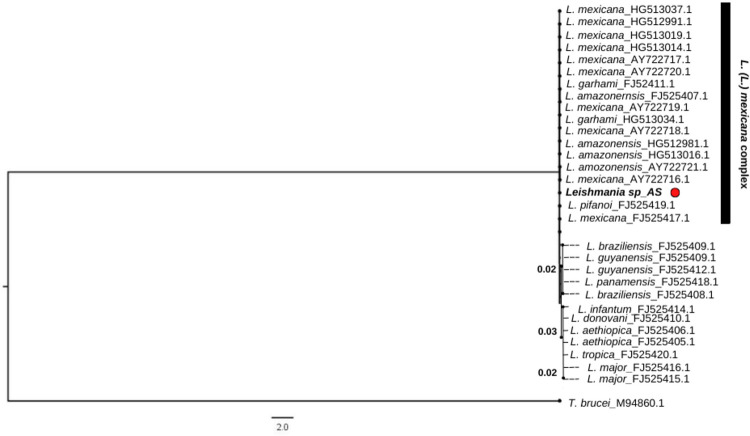
Maximum Likelihood consensus tree based on 7SL RNA gene sequences. Numbers on branches represent bootstrap support values. The sequence derived from asymptomatic subjects in Yucatan is represented by *Leishmania sp*_AS (red dot).

The consensus sequence obtained from two asymptomatic human cases was similar to that of the species of the *L. mexicana* complex (data not shown). The ML analyses based on the 7SL RNA gene did not resolve the phylogenetic relationships between the 12 *Leishmania* species included in this study. Despite the low bootstrap support values obtained in the phylogenetic analysis, the consensus sequence from the asymptomatic subjects was confirmed to be within the clade integrated by the species of the *L. mexicana* complex (Figure 2). Similar results were observed in the bootstrap neighbor-joining tree (data not shown).

## DISCUSSION

In this study, the presence of a persistent infection by *Leishmania* was observed in asymptomatic carriers whose PBMC did not significantly express IFN-γ after *in vitro* stimulation with SLA. The results we obtained are consistent with a previous report from an endemic area of *L. mexicana* transmission in Mexico, in which undetectable levels of IFN-γ expression were found in the MST response of asymptomatic carriers^15^. Similarly, no significant differences were found in IFN-γ production from SLA stimulated-PBMC between asymptomatic and healthy individuals from other CL endemic areas in the Americas^16^. In fact, most studies have revealed that *in vitro* levels of IFN-γ production are lower in asymptomatic subjects than in patients with the overt disease (CL or MCL) or patients cured in response to treatment^4,16^. These findings have raised the question of whether the quantification of this cytokine is reliable for identifying asymptomatic populations. The latter hypothesis was supported by a study that found a moderate agreement between the MST and the production of IFN-γ in identifying exposure to *L. braziliensis* infection in an endemic area of Brazil. In that study, the production of IFN-γ was only observed in 22 (61.1%) of 36 individuals with a positive test^17^.

Another important factor to consider is the exposure time. In this regard, reduced levels of IFN-γ production have been observed in individuals who remained in an endemic area of leishmaniasis for more than 90 days, which indicates that exposure time may be associated with the detection of this cytokine, limiting its use for the identification of asymptomatic infections in people living in endemic areas^7^.

Furthermore, in this study, we observed the presence of the 7SL RNA gene of viable parasites of the *L. mexicana* complex in the blood of subjects with a positive MST from an emerging focus of CL in Yucatan. In the Americas, the amplification of *Leishmania* 7SL RNA gene transcripts has been demonstrated in asymptomatic carriers, revealing their potential as a target for the parasitological diagnosis of asymptomatic infections and overcoming the issue of *Leishmania* DNA detection, which does not differentiate whether the parasite is alive or dead^8^. The detection of this gene has also been suggested to improve the understanding of the role of asymptomatic carriers in the transmission of the disease and in the endemicity of *Leishmania* infection^8^.

Other important implications of the presence of viable parasites in asymptomatic carriers are the possibility of disease activation under immunosuppression and the risk of parasite transmission by blood transfusion^18^. Further studies are urgently needed to dissect the exact role of asymptomatic infections in the *Leishmania* transmission cycle, as this is an important area of knowledge that has been neglected in endemic areas of CL.

Regarding the potential use of the 7SL RNA gene transcripts for the identification of the *Leishmania* species in asymptomatic carriers, in this study, it was not possible to resolve the sequence identity using phylogenetic analyses. All the *L. mexicana* sequences from Mexico included in the study were associated with cutaneous manifestations, but it was not possible to detect potential intraspecific variations with the consensus sequence obtained from asymptomatic subjects using the 7SL RNA gene. An important question that has not been addressed in our country and in the study region is whether *L. mexicana* presents structure and genetic diversity and whether intraspecific variability is related to the outcome of infections in humans. This is important because, in the Old World, it seems that different genotypic units of *L. infantum* are related to asymptomatic or clinical infections^19,20^. Our results highlight the need to use other markers or genetic approaches (metabarcoding/metagenomic analysis) to determine the identity of asymptomatic isolates in Yucatan, as well as to evaluate the genetic variability of the agent causing cutaneous lesions in the region.

## CONCLUSION

We identified a latent infection in asymptomatic individuals whose peripheral blood mononuclear cells did not produce IFN-γ after stimulation with soluble *Leishmania* antigen. The absence of an association between the presence of live *Leishmania* parasites in the blood and the *in vitro* expression of IFN-γ suggests that this cytokine is not a suitable marker of exposure for this population. We suggest further studies including large samples and evaluating more than one cytokine.

## References

[1] World Health Organization Leishmaniasis.

[2] Mannan SB, Elhadad H, Loc TT, Sadik M, Mohamed MY, Nam NH (2021). Prevalence and associated factors of asymptomatic leishmaniasis: a systematic review and meta-analysis. Parasitol Int.

[3] Ibarra-Meneses AV, Corbeil A, Wagner V, Onwuchekwa C, Fernandez-Prada C (2022). Identification of asymptomatic Leishmania infections: a scoping review. Parasit Vectors.

[4] Bittar RC, Nogueira RS, Vieira-Gonçalves R, Pinho-Ribeiro V, Mattos MS, Oliveira-Neto MP (2007). T-cell responses associated with resistance to Leishmania infection in individuals from endemic areas for Leishmania (Viannia) braziliensis. Mem Inst Oswaldo Cruz.

[5] Das VN, Bimal S, Siddiqui NA, Kumar A, Pandey K, Sinha SK (2020). Conversion of asymptomatic infection to symptomatic visceral leishmaniasis: a study of possible immunological markers. PLoS Neg Trop Dis.

[6] Gomes-Silva A, Bittar RC, Nogueira RS, Amato VS, Mattos MS, Oliveira-Neto MP (2007). Can interferon-γ and interleukin-10 balance be associated with severity of human Leishmania (Viannia) braziliensis infection?. Clin Exp Immunol.

[7] Best I, Privat-Maldonado A, Cruz M, Zimic M, Bras-Gonçalves R, Lemesre JL (2018). IFN-γ response is associated to time exposure among asymptomatic immune responders that visited American Tegumentary Leishmaniasis endemic areas in Peru. Front Cell Infect Microbiol.

[8] Rosales-Chilama M, Gongora RE, Valderrama L, Jojoa J, Alexander N, Rubiano LC (2015). Parasitological confirmation and analysis of Leishmania diversity in asymptomatic and subclinical infection following resolution of cutaneous leishmaniasis. PLoS Negl Trop Dis.

[9] Loría-Cervera EN, Sosa-Bibiano EI, Van Wynsberghe NR, Torres-Castro JR, Andrade-Narváez FJ (2019). Preliminary epidemiological findings of Leishmania infection in the municipality of Tinum, Yucatan State, Mexico. Parasite Epidemiol Control.

[10] Loría-Cervera EN, Sosa-Bibiano EI, Van Wynsberghe NR, Saldarriaga OA, Melby PC, Andrade-Narvaez FJ (2016). Cytokine mRNA expression in Peromyscus yucatanicus (Rodentia: Cricetidae) infected by Leishmania (Leishmania) mexicana. Cytokine.

[11] Romero I, Téllez J, Suárez Y, Cardona M, Figueroa R, Zelazny A (2010). Viability and burden of Leishmania in extralesional sites during human dermal leishmaniasis. PLoS Negl Trop Dis.

[12] Simmons MP (2004). Independence of alignment and tree search. Mol Phylogenet Evol.

[13] Hillis DM, Bull JJ (1993). An empirical test of bootstrapping as a method for assessing confidence in phylogenetic analyses. Syst Biol.

[14] Bamorovat M, Sharifi I, Aflatoonian MR, Sadeghi B, Shafiian A, Oliaee RT (2019). Host’s immune response in unresponsive and responsive patients with anthroponotic cutaneous leishmaniasis treated by meglumine antimoniate: a case-control study of Th1 and Th2 pathways. Int Immunopharmacol.

[15] Valencia-Pacheco G, Loría-Cervera EN, Sosa-Bibiano EI, Canché-Pool EB, Vargas-Gonzalez A, Melby PC (2014). In situ cytokines (IL-4, IL-10, IL-12, IFN-γ) and chemokines (MCP-1, MIP-1α) gene expression in human Leishmania (Leishmania) mexicana infection. Cytokine.

[16] Follador I, Araújo C, Bacellar O, Araújo CB, Carvalho LP, Almeida RP (2002). Epidemiologic and immunologic findings for the subclinical form of Leishmania braziliensis infection. Clin Infect Dis.

[17] Schnorr D, Muniz AC, Passos S, Guimaraes LH, Lago EL, Bacellar O (2012). IFN-γ production to leishmania antigen supplements the leishmania skin test in identifying exposure to L. braziliensis infection. PLoS Negl Trop Dis.

[18] França AO, Pereira LO, Tanaka TS, Oliveira MP, Dorval ME (2020). Viability of Leishmania in blood donors: a tangible possibility of transfusion transmission. J Microbiol Immunol Infect.

[19] Rezaei Z, Azarang E, Shahabi S, Omidian M, Pourabbas B, Sarkari B (2020). Leishmania ITS1 is genetically divergent in asymptomatic and symptomatic visceral leishmaniasis: results of a study in Southern Iran. J Trop Med.

[20] Hide M, Marion E, Pomares C, Fisa R, Marty P, Bañuls AL (2013). Parasitic genotypes appear to differ in leishmaniasis patients compared with asymptomatic related carriers. Int J Parasitol.

